# HDAC8 impairs tight junctions in allergic rhinitis through Smad7 deacetylation

**DOI:** 10.3389/fimmu.2026.1793810

**Published:** 2026-05-01

**Authors:** Huan-huan Chang, Zi-ne Cao, Pan-hong Dang, Miao Wei, Yang Li, Hui-ping Zhang, Jie Wang

**Affiliations:** Department of Otolaryngology-Head and Neck Surgery, The Affiliated Children’s Hospital of Xi’an Jiaotong University, Xi’an, Shaanxi, China

**Keywords:** acetylation, allergic rhinitis, epithelial barrier, HDAC inhibitors, histone deacetylase 8, nasal mucosa, Smad7 protein, tight junctions

## Abstract

**Background:**

Allergic rhinitis (AR) involves Th2/Th17 immune dysregulation and nasal epithelial barrier dysfunction; however, the molecular mechanisms linking these processes remain unclear.

**Objective:**

Here, we investigated whether histone deacetylase 8 (HDAC8) contributes to tight junction (TJ) disruption in AR by modulating Smad7 acetylation.

**Methods:**

Primary human nasal epithelial cells and a house dust mite-induced AR mouse model were used in the study. HDAC8 was modulated via overexpression, siRNA knockdown, or pharmacological inhibition (PCI-34051). Smad7 function was assessed using wild-type and acetylation-mimetic (K64/70Q) mutants. Protein interactions and expression levels of HDAC8, Smad7, acetyl-Smad7, and TJ proteins (ZO-1, Occludin, Claudin-1) were analyzed by co-immunoprecipitation and western blot. Nasal pathology and serum cytokines were evaluated by histology and ELISA, respectively.

**Results:**

In AR mouse models, HDAC8 expression was upregulated and showed enhanced binding to Smad7, accompanied by reduced Smad7 acetylation and decreased TJ protein expression. PCI-34051 treatment or HDAC8 knockdown restored Smad7 acetylation and TJ protein expression. Notably, the acetylation-mimetic Smad7 mutant rescued HDAC8-induced TJ disruption, suggesting that Smad7 acetylation status is involved in this effect. *In vivo*, PCI-34051 treatment improved nasal epithelial integrity and modulated Th1/Th2/Th17/Treg-associated cytokine profiles.

**Conclusion:**

These findings support that HDAC8-mediated reduction of Smad7 acetylation contributes to epithelial barrier dysfunction in AR, highlighting HDAC8 as a potential therapeutic target for restoring mucosal homeostasis.

## Introduction

Allergic rhinitis (AR) is a non-infectious disease of the nasal mucosa mediated primarily by immunoglobulin E (IgE) following allergen exposure (1), affecting nearly 40% of the global population (2). Although various treatment options are available clinically-including stepped pharmacotherapy, allergen-specific immunotherapy, traditional Chinese medicine, and surgical intervention-an international, multicenter, cross-sectional study revealed that a substantial proportion of AR patients remain dissatisfied with symptom control (3). Thus, despite existing therapies, the need for more effective long-term treatment options persists.

Epigenetic modifications, including histone acetylation, have been implicated in the pathogenesis of AR (4). Lysine acetylation of histone and non-histone proteins is a reversible post-translational modification that influences gene expression patterns. This process is dynamically regulated by the opposing actions of histone acetyltransferases, which add acetyl groups to lysine residues, and histone deacetylases (HDACs), which remove them (5). Beyond their classical role in histone modification, HDACs can also target non-histone proteins. Notably, HDAC1, -2, -3, and HDAC5, -6 have been shown to deacetylate Smad7 (6). Smad7 can negatively regulate transforming growth factor-beta (TGF-β) signal transduction and also regulate stem cell self-renewal and differentiation in a TGF-β-independent manner (7).

Epithelial tight junctions (TJs) are macromolecular complexes composed of transmembrane proteins (e.g., Occludin, JAMs, Claudins) and cytoplasmic plaque proteins (e.g., Zonula Occludens, ZOs). These structures form a continuous intercellular network that seals the paracellular space and maintains epithelial barrier integrity (8). In nasal mucosal epithelial cells, TJs not only provide a physical barrier but also help regulate immune responses to pathogens and allergens (9). Even in the absence of inflammatory mediators, a defective nasal mucosal epithelial barrier can directly contribute to mast cell degranulation and allergic sensitization (10). Epithelial cells cultured *in vitro* from patients for several weeks still show decreased epithelial integrity, suggesting a potential epigenetic basis for this “memory” effect (11).

**Figure 1:**
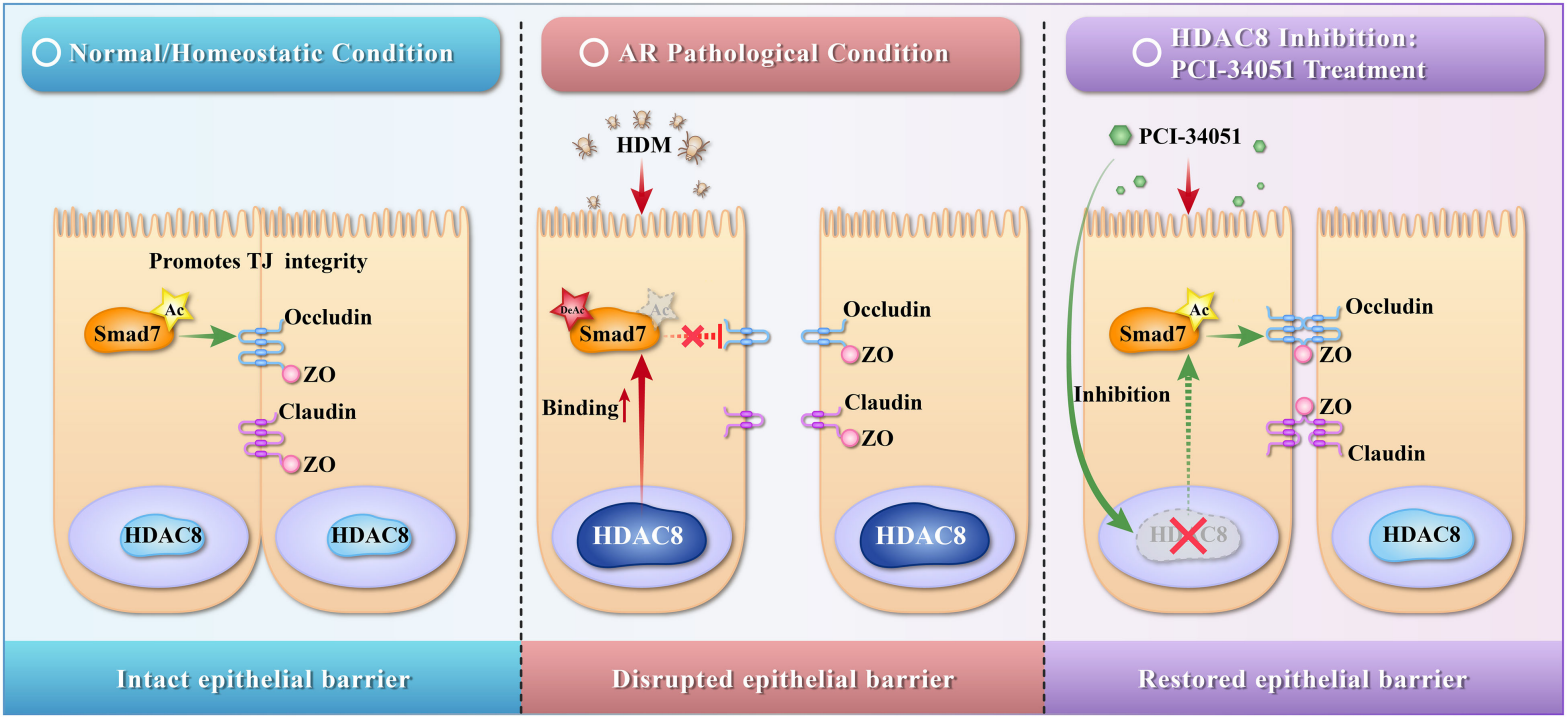
Schematic diagram of the proposed HDAC8-Smad7-TJ axis in AR pathogenesis. Under normal physiological conditions (left panel), basal HDAC8 activity allows Smad7 to maintain its acetylated state, supporting TJ integrity and epithelial barrier function. In allergic rhinitis (AR, middle panel), allergen exposure upregulates HDAC8 expression and enhances its binding to Smad7. This leads to Smad7 deacetylation, which impairs Smad7-mediated TJ protection, resulting in disrupted TJ structures and compromised epithelial barrier integrity. Treatment with the HDAC8-specific inhibitor PCI-34051 (right panel) blocks HDAC8 activity, restores Smad7 acetylation, and rescues TJ protein expression, thereby promoting epithelial barrier restoration. This diagram represents a working model based on the current findings; dashed lines and arrows indicate relationships that are supported by our data but may involve additional, yet-to-be-identified intermediate steps. TJ, tight junction; Ac, acetylation; DeAc, deacetylation; HDM, house dust mite; →, activation or promotion; →, inhibition or disruption. This image was created using BioRender (BioRender.com, Toronto, Canada).

Our previous studies demonstrated that HDAC8 expression is increased in the nasal tissue of ovalbumin-sensitized AR mice. Non-specific HDAC inhibitors reduced HDAC8 expression (12), decreased behavioral scores and serum ovalbumin-IgE levels in AR mice, indicating therapeutic potential (13). Literature reports the expression of Smad7 was upregulated in the nasal tissue of AR mice (14). Based on these observations, we hypothesize that following allergen exposure, elevated HDAC8 in nasal mucosal epithelial cells binds to Smad7 and reduces its acetylation, thereby impairing Smad7-mediated regulation of TJ and contributing to epithelial barrier dysfunction in AR (**Figure 1**).

In this study, we employed human primary nasal epithelial cells (PNECs) cultured at air-liquid interface (ALI) and a house dust mite (HDM)-sensitized AR mouse model to investigate the HDAC8/Smad7 acetylation/TJ axis in AR pathogenesis. Elucidating this mechanism may provide a foundation for developing more targeted therapeutic strategies for AR.

## Materials and methods

### Reagents, antibodies, and constructs

All reagents, antibodies, plasmids, and constructs used in this study are listed in **Supplementary Table S1**.

### Air-liquid interface cultures

Normal nasal mucosal tissues were collected from patients undergoing nasal septum deviation correction or optic nerve decompression at the affiliated Children’s Hospital of Xi’an Jiaotong University (Xi’an, Shaanxi Province, China). All donors were confirmed to have no history of nasal inflammation or allergic diseases. The age range of enrolled patients was 15 to 18 years, and sample collection took place between January 2023 and December 2023. Cells from three independent donors were used for each experimental condition (n = 3) (Table 1).

**Table 1:** Human donor characteristics (PNEC Sources).

Donor					
ID	Age (years)	Sex	Diagnosis	Tissue Source	Collection Date
H01	16	M	Nasal septum deviation	Inferior turbinate	2023-01
H02	15	F	Nasal septum deviation	Inferior turbinate	2023-08
H03	18	M	Optic nerve decompression	Middle turbinate	2023-12

All donors were confirmed to have no history of nasal inflammation or allergic diseases. F, female; M, male; PNEC, primary nasal epithelial cell.

Tissues were obtained as surgical biopsies (~3–5 mm³) from the inferior or middle turbinate during routine procedures. Immediately after collection, tissue specimens were placed in ice-cold sterile phosphate-buffered saline containing 1% penicillin-streptomycin and transported to the laboratory within 30 minutes. For cell isolation, tissues were washed three times with phosphate-buffered saline, minced into approximately 1 mm³ fragments, and digested with 0.1% protease XIV (Sigma-Aldrich, St. Louis, MO, USA) in Dulbecco’s Modified Eagle Medium (DMEM) overnight at 4 °C. The digestion was stopped by adding 10% fetal bovine serum, and the cell suspension was filtered through a 100 μm cell strainer. Isolated cells were centrifuged at 300 × g for 10 minutes, resuspended in culture medium, and seeded into collagen-coated flasks.

Cells were cultured in bronchial epithelial basal medium (BEBM, Lonza, Basel, Switzerland) supplemented with growth factors according to the manufacturer’s instructions (Lonza SingleQuots™, excluding retinoic acid) and mixed with Dulbecco’s Modified Eagle Medium (GIBCO BRL, Invitrogen, Carlsbad, CA) at a 1:1 ratio. Fresh all-trans retinoic acid (Sigma-Aldrich, Saint Louis, MO) was added to the culture medium at a final concentration of 10 ng/mL. When cells reached 80-90% confluence in monolayer culture, they were detached using 0.25% trypsin-EDTA (GIBCO BRL) and seeded onto 12-well Transwell^®^ permeable supports (0.4 μm pore size, polyester membrane; Corning, NY, USA) at a density of 1 × 10^5^ cells per well. Cells were maintained in submerged culture with medium added to both apical (0.5 mL) and basolateral (1.5 mL) compartments. Medium was replaced every 48 hours until cells reached confluence. Upon reaching confluence (typically 5–7 days after seeding), an ALI was established by removing the apical medium and providing fresh medium only to the basolateral compartment (1.5 mL). Cells were maintained at ALI for 21 days to promote mucociliary differentiation, with basolateral medium changed every 48 hours. All cultures were maintained at 37 °C in a humidified atmosphere containing 5% CO_2_. Differentiation was monitored by assessing the appearance of beating cilia and mucus production under phase-contrast microscopy. Fully differentiated cultures were used for subsequent experiments as described in the main text. Cells were stimulated with 100 μg/mL HDM extract for 24 h to establish an *in vitro* AR model (15).

### Animals and treatment protocol

A total of 30 female BALB/c mice, aged 6 weeks and weighing 18–20 g, were purchased from Xi’an Jiaotong University. Mice were raised in a pathogen-free environment under a 12 h light/12 h dark cycle at 22 °C with free access to food and water. The animals were randomly divided into three experimental groups (n=10 per group): a control group, an AR model group, and a PCI-34051 treatment group. Mice were randomly assigned to experimental groups using a computer-generated random number sequence. Randomization was performed by an investigator not involved in subsequent experimental procedures. Blinding was implemented as follows: symptom scoring was conducted by an independent researcher unaware of group allocation; histological and Western blot (WB) analyses were performed and quantified by investigators blinded to treatment groups. No animals were excluded from the analysis. All experimental procedures were conducted in accordance with ARRIVE 2.0 guidelines.

The HDM sensitization and challenge protocol was adapted from previously described methods (11, 28). This model induces eosinophilic inflammation mediated by a type 2 immune response triggered by HDM. BALB/c mice were endonasally sensitized with 50 μL HDM extract (1μg, Greer Laboratories, Lenoir, NC) or exposed to 50 μL of saline at day 1. From days 8 to 12, mice were endonasally challenged with 50μL HDM extract (10 μg/day) or saline. This regimen is identical to that described in references (11, 28), except that the cited studies used C57BL/6 mice, whereas we used BALB/c mice. One hour prior to each HDM administration, mice were intraperitoneally injected with either the HDAC8 inhibitor [PCI-34051 (0.5 mg/kg)] or 0.02 mL of 50% dimethyl sulfoxide (vehicle control). Following the final challenge, behavioral scores were assessed for each animal over a 30-minute observation period. Under anesthesia induced by intraperitoneal injection of 4% chloral hydrate (400 mg/kg), blood samples (approximately 100-150 μL) were collected via the orbital plexus. The mice were then euthanized by cervical dislocation, and nasal tissues were harvested and immediately frozen in liquid nitrogen for subsequent analysis.

### Ethical statement and humane endpoints

All animal experiments were approved by the Institutional Animal Care and Use Committee of Xi’an Jiaotong University (No.2020-447). Humane endpoints included: (1) >20% body weight loss; (2) severe respiratory distress lasting >1 hour post-challenge; (3) inability to ambulate normally for >12 hours; (4) self-injurious behavior causing tissue damage. Upon attainment of any humane endpoint, animals were immediately euthanized by cervical dislocation to ensure rapid and humane termination.

### Histological analysis

Following fixation in 4% paraformaldehyde, nasal tissues were paraffin-embedded and sectioned. For histological analysis, deparaffinized sections were either stained with Alcian Blue (pH 2.5) and counterstained with Nuclear Fast Red or processed for routine hematoxylin and eosin staining. All stained sections were visualized using a standard bright-field microscope (Olympus BX53, Tokyo, Japan).

### Western blot analysis

Total protein was extracted from cells or nasal mucosal tissues using a commercial lysis buffer (Applygen Technologies, Beijing, China) supplemented with protease and phosphatase inhibitor cocktails (Roche, Basel, Switzerland). Protein concentrations were determined using the Bio-Rad Protein Assay Kit (Bio-Rad Laboratories, Hercules, CA, USA) according to the manufacturer’s instructions. Equal amounts of protein (30 μg per lane) were separated by sodium dodecyl sulfate-polyacrylamide gel electrophoresis on 12.5% polyacrylamide gels and electrophoretically transferred to nitrocellulose membranes (0.45 μm pore size; Bio-Rad). Membranes were blocked with 2.5% non-fat milk in Tris-buffered saline containing 0.1% Tween-20 (TBST) for 1 hour at 37 °C, then incubated overnight at 4 °C with primary antibodies diluted in TBST containing 1% bovine serum albumin. The following primary antibodies were used: HDAC8, ZO-1, Occludin, Claudin-1, Smad7 and glyceraldehyde-3-phosphate dehydrogenase (GAPDH). After three 10-minute washes with TBST, membranes were incubated with HRP-conjugated secondary antibodies at 37 °C for 1 hour. Protein bands were visualized using an enhanced chemiluminescence detection system (SuperSignal West Pico PLUS, Thermo Fisher Scientific) and imaged with a ChemiDoc XRS + system (Bio-Rad). Band intensities were quantified using ImageJ software (National Institutes of Health, Bethesda, MD, USA) and normalized to GAPDH as the loading control. All WB experiments were performed in triplicate using independent biological samples.

### Co-immunoprecipitation analysis

Cells or nasal mucosal tissues were lysed in ice-cold IP lysis buffer (25 mM Tris-HCl pH 7.4, 150 mM NaCl, 1% NP-40, 5% glycerol, 1 mM EDTA; Thermo Fisher Scientific) freshly supplemented with protease and phosphatase inhibitor cocktails (Roche). Lysates were incubated on ice for 30 minutes with intermittent vortexing, followed by centrifugation at 14,000 × g for 15 minutes at 4 °C to remove insoluble debris. Protein concentrations were determined using the Bio-Rad DC Protein Assay Kit. For each IP reaction, 500 μg of total protein was pre-cleared by incubation with 20 μL of Protein A/G agarose beads (Santa Cruz Biotechnology, Dallas, TX, USA) for 1 hour at 4 °C with gentle rotation. Pre-cleared lysates were then incubated with 2 μg of primary antibody (rabbit anti-HDAC8 and rabbit anti-Smad7) or an equal amount of normal rabbit IgG (sc-2027, Santa Cruz Biotechnology) as a negative control, overnight at 4 °C with constant rotation. The following day, 40 μL of Protein A/G agarose beads were added to each sample and incubated for an additional 2 hours at 4 °C with rotation. Beads were collected by centrifugation at 1,000 × g for 1 minute at 4 °C and washed five times with 500 μL of ice-cold IP lysis buffer to remove non-specifically bound proteins. Immunoprecipitated proteins were eluted by resuspending beads in 40 μL of 2× Laemmli sample buffer (Bio-Rad) containing 5% β-mercaptoethanol and boiling at 95 °C for 5 minutes. The eluted samples were then subjected to WB analysis as described above, using appropriate antibodies to detect target proteins and their acetylated forms. For detection of acetylated Smad7, membranes were probed with anti-acetylated-lysine antibody followed by stripping and reprobing with anti-Smad7 antibody to confirm equal IP efficiency. Input controls (10% of lysate used for IP) were included in all experiments to verify equal starting protein levels.

### Plasmid construction and cell transfection

The full-length cDNA encoding human HDAC8 (NM_018486.3) was amplified by PCR and cloned into the pcDNA3.1(+) mammalian expression vector (Invitrogen, Carlsbad, CA, USA) between the HindIII and EcoRI restriction sites to generate the HDAC8 overexpression (HDAC8-OE) plasmid (pcDNA3.1-HDAC8). The wild-type (WT) Smad7 plasmid (pcDNA3.1-Smad7-WT) was constructed by inserting the full-length human Smad7 cDNA (NM_005904.4) into the same vector at the BamHI and XhoI sites. To generate the acetylation-mimetic Smad7 mutant (Smad7-KQ), site-directed mutagenesis was performed using the QuikChange II XL Site-Directed Mutagenesis Kit (Agilent Technologies, Santa Clara, CA, USA) according to the manufacturer’s protocol. Both known acetylation sites (Lys64 and Lys70) were simultaneously substituted with glutamine (K64Q and K70Q) using the following primer pairs: forward 5’-…-3’ and reverse 5’-…-3’ (sequences available upon request). All plasmid constructs were verified by Sanger DNA sequencing (Tsinke Biotechnology, Xi’an, China) to confirm the correct sequences and the presence of desired mutations.

For transfection experiments, PNECs were seeded in 6-well plates at a density of 2×10^5^ cells per well and cultured until reaching 70-80% confluence. Cells were transfected with plasmid DNA using Lipofectamine 3000 transfection reagent (Invitrogen) according to the manufacturer’s instructions. For each well, 2.5 μg of total plasmid DNA and 5 μL of Lipofectamine 3000 were diluted in Opti-MEM reduced serum medium (Gibco, Grand Island, NY, USA). For co-transfection experiments, plasmids were mixed at a 1:1 ratio (1.25 μg each) to a total of 2.5 μg DNA per well. The empty pcDNA3.1(+) vector was used to supplement the total DNA amount to ensure consistency across all transfection conditions. The DNA-lipid complexes were added to cells and incubated for 6 hours, after which the medium was replaced with fresh complete culture medium. Cells were harvested for subsequent analysis 24 hours post-transfection. Transfection efficiency was assessed in parallel wells using a GFP-expressing plasmid and was consistently >60% across all experiments. All transfection experiments were performed in triplicate with independent biological replicates.

### Statistical analysis

Statistical analyses were performed using SPSS 13.0 and GraphPad Prism 8.0. Data are presented as mean ± standard deviation (SD). For *in vitro* experiments, at least three independent biological replicates were performed, each with three technical replicates. For animal experiments, each mouse was considered an independent biological replicate, with n=10 per group. Normality was assessed by Shapiro-Wilk test and homogeneity of variances by Levene’s test. Multiple group comparisons were analyzed by one-way analysis of variance followed by Fisher’s least significant difference (LSD) *post hoc* test when variances were equal, or Brown-Forsythe analysis of variance with Games-Howell test when variances were unequal. Two-group comparisons were analyzed by unpaired two-tailed Student’s t-test. A value of P < 0.05 was considered statistically significant.

## Results

The experimental design and main outcomes are summarized in **Table 2**.

**Table 2:** Experimental groups, interventions, and primary outcome measures.

Experiment	Groups	Interventions	Primary outcomes
*In vitro* (**Figure 2**)	Vehicle Control; HDM; HDM + PCI; HDM + si-HDAC8; Empty vector control; HDAC8-OE	PCI-34051 (50 μM); si-HDAC8; HDAC8 plasmid; HDM (100μg/mL)	Smad7 acetylation; ZO-1, Occludin, Claudin-1 expression
*In vitro* (**Figure 3**)	Empty vector control; HDAC8-OE; HDAC8-OE + Smad7 (WT); HDAC8-OE + Smad7 (KQ)	HDAC8 plasmid; Smad7-WT; Smad7-KQ	ZO-1, Occludin, Claudin-1 expression
*In vivo* (**Figures 4**–**6**)	Vehicle Control; AR; AR +PCI (n=10/group)	HDM sensitization (1 μg) and Challenge (10 μg/d); PCI-34051 (0.5 mg/kg i.p.)	Symptom scores; HDM-IgE; Serum cytokines (IL-2, IL-4, IL-17, TGF-β1); histology; Smad7 acetylation; TJ protein expression

AR, allergic rhinitis; HDM, house dust mite; HDAC8, histone deacetylase 8; i.p., intraperitoneal; OE, overexpression; PCI-34051, HDAC8-specific inhibitor; si-HDAC8, small interfering RNA targeting HDAC8; Smad7 (KQ), acetylation-mimetic Smad7 mutant (K64/70Q); TJ, tight junction; WT, wild-type.

### HDAC8 modulates Smad7 acetylation and TJ expression in PNECs

**Figure 2:**
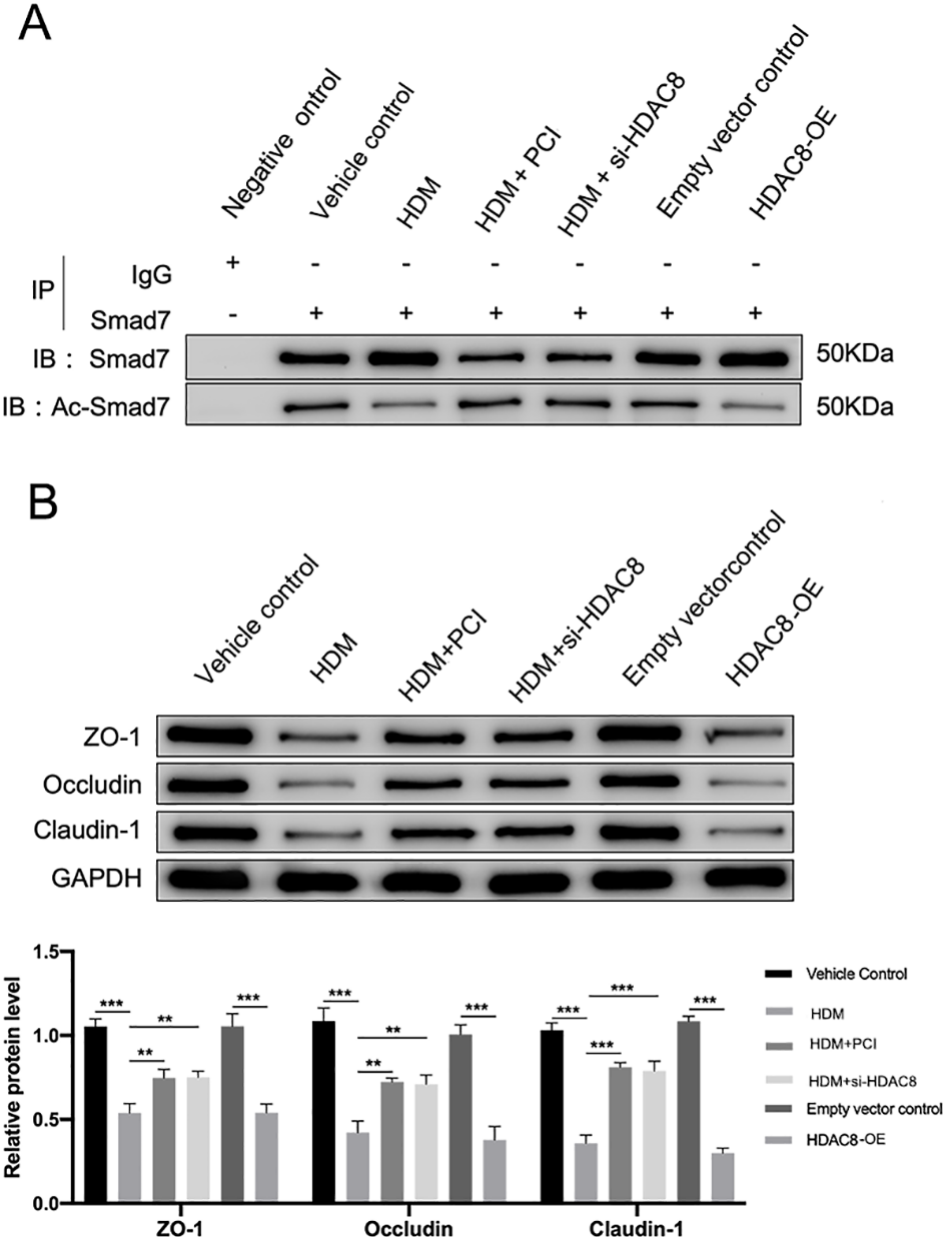
AR/HDAC8 induce epithelial barrier dysfunction via Smad7 deacetylation. **(A)** Co-IP analysis of Smad7 acetylation in PNECs under indicated conditions. Cell lysates were immunoprecipitated with anti-Smad7 antibody or control IgG, followed by immunoblotting for total Smad7 and Ac-Smad7. No Smad7 or Ac-Smad7 signals were detected in the IgG control, confirming antibody specificity. Compared with vehicle and empty vector controls, HDM treatment or HDAC8-OE increased total Smad7 levels while decreasing Ac-Smad7 levels. Conversely, HDAC8 inhibition with PCI-34051 (HDM+PCI) or HDAC8 knockdown with siRNA (HDM+si-HDAC8) reversed these effects, reducing total Smad7 and restoring Ac-Smad7 to baseline levels. **(B)** Representative WB and quantitative analysis of TJ proteins ZO-1, Occludin, and Claudin-1. HDM treatment or HDAC8-OE significantly reduced the expression of all three TJ proteins compared with controls. These reductions were abrogated by PCI-34051 treatment or HDAC8 knockdown, which restored protein levels to near-baseline. Data are presented as mean ± SD. **P < 0.01, ***P < 0.001 versus indicated controls.

To investigate the role of HDAC8 in regulating Smad7 acetylation and TJ expression, we performed experiments using PNECs cultured under AR-mimicking conditions (HDM stimulation) or with HDAC8 modulation. In Co-IP assays (**Figure 2A**), HDM treatment or HDAC8-OE was associated with increased total Smad7 protein levels and reduced acetylated Smad7 (Ac-Smad7) compared with controls. Conversely, HDAC8 inhibition with PCI-34051 or small interfering RNA (siRNA) knockdown decreased total Smad7 and restored Ac-Smad7 levels. These observations suggest a relationship between HDAC8 activity and Smad7 acetylation status. Further WB analysis of TJ (**Figure 2B**) demonstrated that HDM treatment and HDAC8-OE also significantly downregulated the expression of ZO-1, Occludin, and Claudin-1. Similarly, HDAC8 inhibition or knockdown reversed this effect, restoring the expression of these proteins to near-baseline levels.

Taken together, these findings demonstrate that in PNECs, HDAC8 modulates Smad7 acetylation and TJ expression, establishing Smad7 deacetylation as a key mechanistic link between AR signaling, HDAC8 upregulation, and epithelial barrier dysfunction.

### Smad7 acetylation status determines susceptibility to HDAC8-induced TJ disruption in PNECs

**Figure 3:**
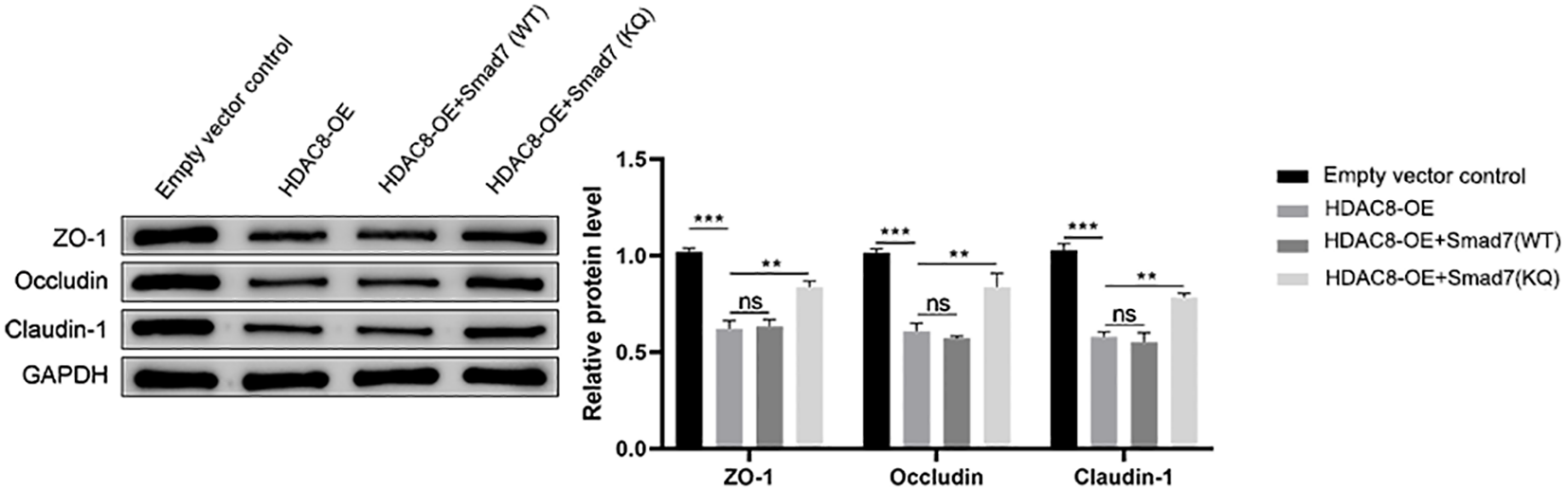
Smad7 acetylation is critical for protecting TJs against HDAC8-induced disruption. PNECs were transfected with indicated plasmid combinations. Representative WB images and quantitative analysis of ZO-1, Occludin, and Claudin-1 protein levels are shown. Compared with empty vector control, HDAC8-OE significantly decreased expression of all three TJ proteins. Co-expression of HDAC8-OE with Smad7 (WT) did not significantly rescue this effect. In contrast, co-expression of HDAC8-OE with Smad7 (KQ) significantly restored TJ protein expression. Data are presented as mean ± SD. **P < 0.01, ***P < 0.001 versus indicated groups.

To determine whether Smad7 acetylation mediates HDAC8-induced TJ changes, we conducted rescue experiments in PNECs using wild-type (WT) and acetylation-mimetic Smad7 constructs, and designed an *in vitro* experiment consisting of four groups: empty vector control group, HDAC8-OE group, HDAC8-OE + Smad7 (WT) group, and HDAC8-OE + Smad7 (KQ) group. To mimic the constitutively acetylated state, we generated a Smad7 (KQ) mutant plasmid by substituting both known acetylation sites (Lys64 and Lys70, designated K64/70Q) with glutamine. Subsequently, the HDAC8-OE plasmid was co-transfected with either the Smad7 (WT) or Smad7 (KQ) mutant plasmid into cells. Our results demonstrated that: compared to the empty vector control group, the HDAC8-OE group exhibited significant TJ damage. Co-expression of HDAC8-OE with Smad7 (WT) failed to rescue this TJ injury. In contrast, co-expression of HDAC8-OE with Smad7 (KQ) significantly attenuated HDAC8-induced TJ protein reduction (**Figure 3**), indicating that Smad7 acetylation status modulates susceptibility to HDAC8-mediated effects.

### HDAC8 inhibition attenuates allergic responses and nasal mucosal pathology in HDM-induced AR mice

**Figure 4:**
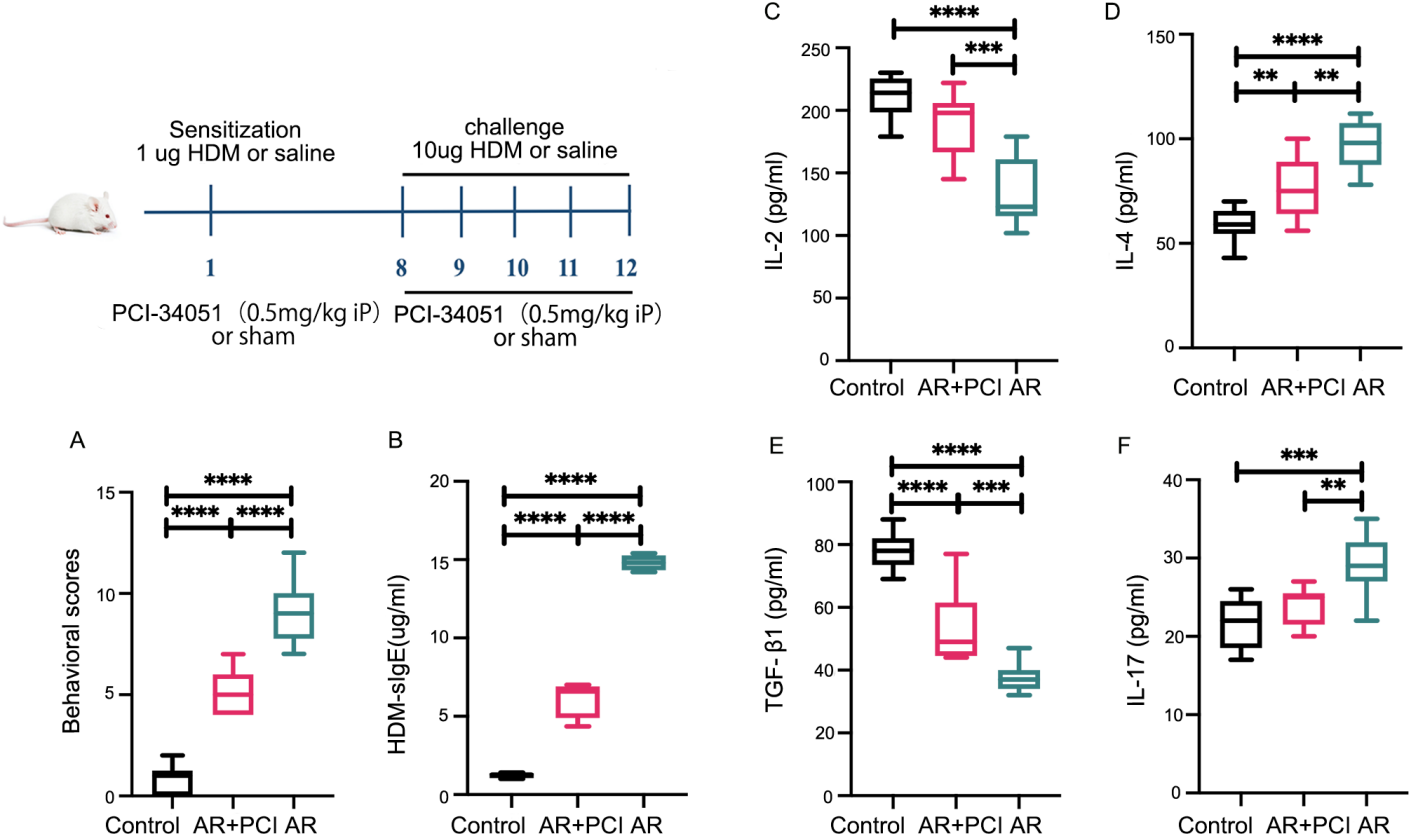
HDAC8 inhibition attenuates allergic responses and modulates serum cytokine profiles in AR mice. **(A)** Allergic symptom scores and **(B)** serum levels of HDM-specific IgE measured by ELISA in control, AR model, and PCI-34051-treated (AR+PCI) groups. **(C–F)** Serum levels of **(C)** IL-2, **(D)** IL-4, **(E)** TGF-β1, and **(F)** IL-17 measured by ELISA. PCI-34051 treatment significantly reduced symptom scores and HDM-specific IgE levels compared with the AR group. For cytokine profiles, AR mice showed decreased IL-2 and TGF-β1 with increased IL-4 and IL-17; PCI-34051 treatment partially reversed these alterations. Data are presented as mean ± SD, **p < 0.01, ***p < 0.001, ****p < 0.0001 versus indicated groups.

**Figure 5:**
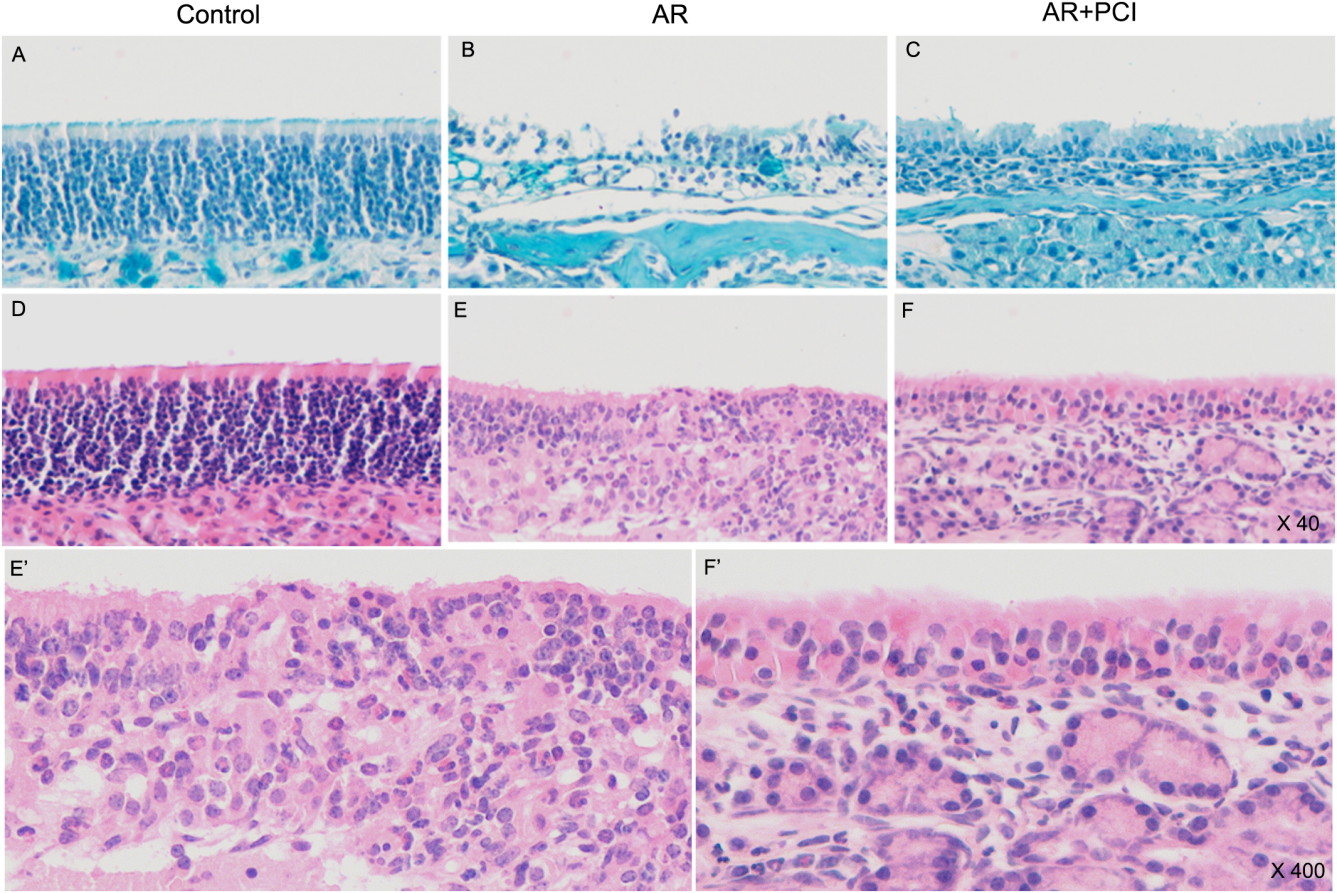
HDAC8 inhibition ameliorates nasal mucosal pathology in AR mice. Representative histological images of nasal mucosa sections stained with Alcian Blue **(A–C)** and hematoxylin and eosin **(A, D–F)** Control group: Epithelial cells are neatly arranged with minimal mucin secretion at the apical surface **(A)**. No eosinophil infiltration is observed **(D)**. **(B, E)** AR group: Marked epithelial cell loss, submucosal edema, and vascular dilation are evident **(B)**. Eosinophil infiltration is visible in both epithelial and submucosal layers **(E)**. **(C, F)** PCI-34051-treated group: Cilia loss is reduced compared with AR group, with increased mucin secretion **(C)**. Mild eosinophil infiltration persists but is substantially reduced **(E', F')** Higher-magnification views of **(E, F)**, respectively, showing eosinophils characterized by lobulated nuclei and cytoplasmic eosinophilic granules.

To validate the *in vivo* relevance of our findings, we employed a HDM-induced AR mouse model. The experiment included three groups: a Control group, an AR model group, and an AR+PCI group. Our results demonstrated that: PCI-34051 treatment was associated with reduced symptom scores and HDM-specific IgE levels (**Figures 4A, B**), as well as modulated serum levels of cytokines associated with Th1 (interleukin-2, IL-2), Th2 (IL-4), Th17 (IL-17), and Treg (TGF-β1) pathways (**Figures 4C–F**). Furthermore, mice treated with PCI-34051 exhibited significant mitigation of nasal mucosal pathology compared to the AR group, including reduced cilia loss, submucosal edema, vascular hyperplasia, mucin secretion, and eosinophil infiltration (**Figure 5**).

### HDAC8 mediates nasal mucosal barrier damage through Smad7 deacetylation in AR

**Figure 6:**
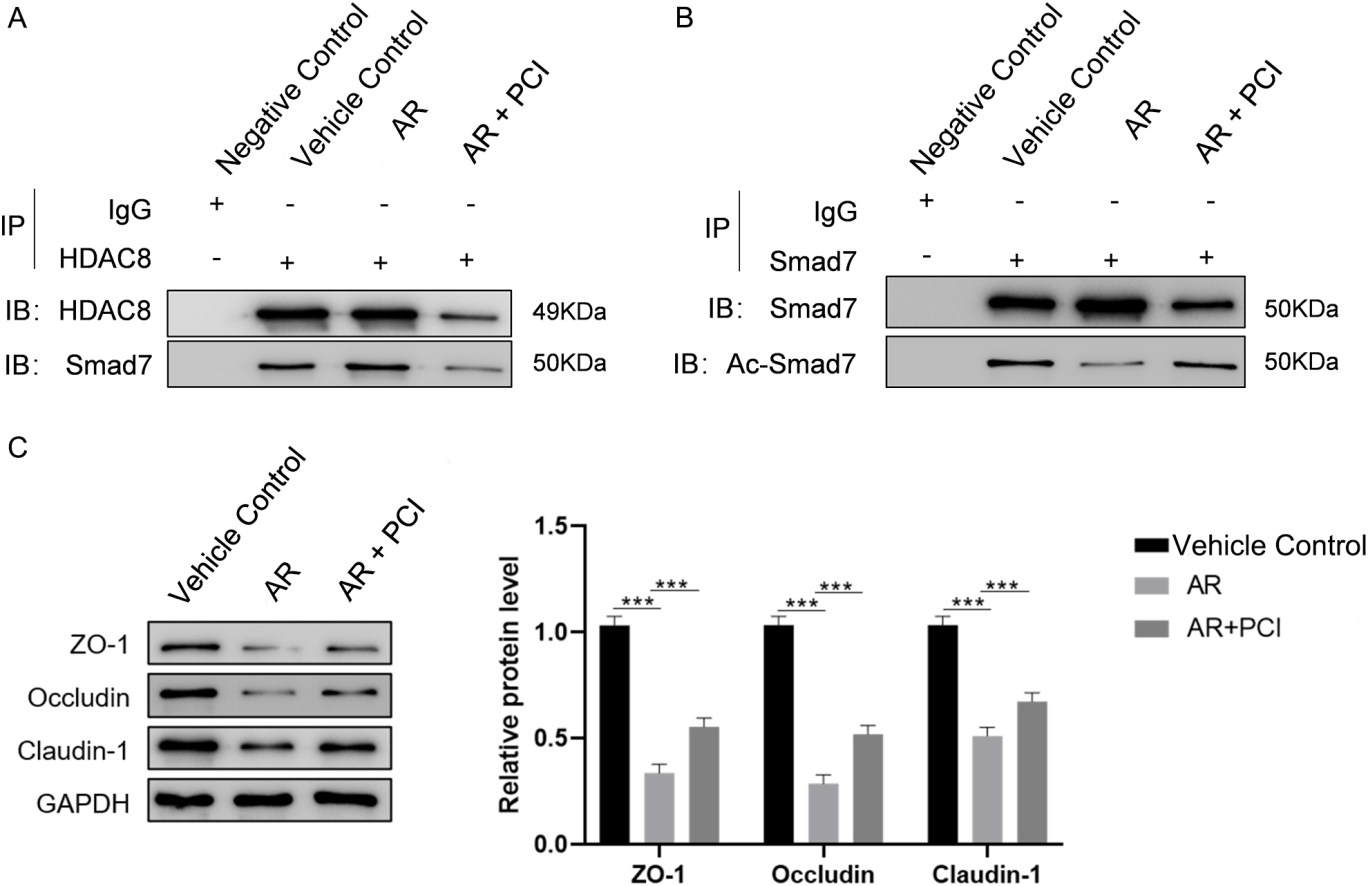
HDAC8 inhibition restores Smad7 acetylation and TJ integrity by disrupting HDAC8-Smad7 interaction in AR mice. **(A)** Co-IP analysis of HDAC8-Smad7 interaction in nasal mucosal tissues. Tissue lysates were immunoprecipitated with anti-HDAC8 antibody or control IgG, followed by immunoblotting for Smad7. Enhanced HDAC8-Smad7 binding was observed in AR mice compared with vehicle controls, which was disrupted by PCI-34051 treatment. **(B)** Co-IP analysis of Smad7 acetylation status. Tissue lysates were immunoprecipitated with anti-Smad7 antibody, followed by WB for total Smad7 and Ac-Smad7. AR mice showed increased total Smad7 but reduced Ac-Smad7 levels compared with controls; PCI-34051 treatment reversed these changes. **(C)** Representative WB and quantitative analysis of TJ proteins ZO-1, Occludin, and Claudin-1 in nasal mucosal tissues. AR-induced reductions in all three proteins were significantly restored by PCI-34051 treatment. Data are presented as mean ± SD. ***P < 0.001 versus indicated groups. Data are presented as mean ± SD, ***p < 0.001.

To confirm the HDAC8-Smad7 interaction and its functional consequences *in vivo*, we performed Co-IP and WB analyses on nasal mucosal tissues from AR mice. Co-IP analysis revealed enhanced HDAC8-Smad7 binding in AR tissues compared with controls, which was reduced by PCI-34051 treatment (**Figure 6A**). These findings suggest that AR conditions are associated with increased HDAC8-Smad7 interaction, and that this association can be modulated by HDAC8 inhibition. Assessment of Smad7 acetylation status showed increased total Smad7 but reduced Ac-Smad7 in AR tissues, effects that were reversed by PCI-34051 treatment (**Figure 6B**). Furthermore, WB analysis of TJ demonstrated that AR-induced reductions in ZO-1, Occludin, and Claudin-1 expression were significantly restored by PCI-34051 treatment (**Figure 6C**), consistent with the protective effect of HDAC8 inhibition on epithelial barrier integrity.

## Discussion

The typical pathological features of AR are characterized by the coexistence of Th2/Th17 immune dysregulation and nasal epithelial barrier dysfunction. Although inflammatory cytokines are known to disrupt TJs, the epigenetic and post-translational mechanisms that translate immune signals into structural epithelial damage remain unclear. Based on their homology to yeast proteins, HDACs are categorized into four classes: Class I (HDAC1, -2, -3, -8), Class IIa (HDAC4, -5, -7, -9), Class IIb (HDAC6, -10), and Class IV (HDAC11), with Class III comprising sirtuins. Among them, HDAC8 exhibits the highest enzymatic activity (HDAC8 > HDAC1 > HDAC3 > HDAC6), shares a catalytic site structurally similar to yeast Rpd3, and is predominantly localized in the nucleus (6). Upregulation of HDAC8 has been observed in lung tissues of ovalbumin-sensitized murine asthma models (16), and PCI-34051 has been shown to mitigate eosinophil-mediated inflammation and airway hyperresponsiveness in such models (17). However, whether and how HDAC8 specifically regulates epithelial barrier integrity in AR, and what its direct downstream targets are, remain to be elucidated.

Smad7 undergoes acetylation via interaction with the acetyltransferase p300 at both its N- and C-terminal regions (6). Deacetylation of Smad7 compromises its protein stability (18), while non-specific HDAC inhibitors help maintain Smad7 expression by preventing its deacetylation (19, 20). In AR, Smad7 mRNA expression is downregulated in nasal tissues of AR rats (21), whereas its overexpression alleviates TNF-α-induced suppression of ZO-1 and Occludin in intestinal epithelial cells (22), underscoring its role in epithelial barrier regulation. TJ integrity is maintained by ZO proteins, which link transmembrane TJ components and recruit transcriptional regulators (23). Occludin modulates paracellular permeability (23), and Claudin-1, which is expressed in human nasal mucosa (24), determines TJ selectivity and strength (25). AR pathogenesis involves nasal mucosal abnormalities such as mucus hypersecretion, edema, goblet cell hyperplasia, epithelial injury, and barrier dysfunction (26). Consistently, reduced TJ expression, including Occludin and ZO-1, has been documented in both AR patients (27) and HDM-sensitized AR animal models (28). Furthermore, IL-4 stimulation downregulates the expression of Occludin and ZO-1 in murine nasal epithelium, and the extent of barrier disruption correlates positively with AR symptom severity (29). Notably, pan-HDAC inhibitors can attenuate HDAC activity and restore expression of TJ-related proteins such as Occludin, ZO-1, and Claudin-7, thereby promoting epithelial barrier repair (11). This suggests that HDACs may serve as a critical node linking inflammation to barrier damage by regulating the acetylation status of Smad7.

Based on these observations, we investigated whether HDAC8 contributes to TJ disruption in AR by modulating Smad7 acetylation. Our findings demonstrate that AR conditions and HDAC8-OE are associated with decreased Smad7 acetylation and reduced TJ protein expression, effects that were mitigated by HDAC8 inhibition or knockdown. Co-IP assays revealed enhanced HDAC8-Smad7 binding in AR tissues, suggesting an association between these proteins under inflammatory conditions. Notably, the acetylation-mimetic Smad7 mutant (K64/70Q) attenuated HDAC8-induced TJ changes, indicating that Smad7 acetylation status may influence susceptibility to HDAC8-mediated effects. Together, these data support a model in which HDAC8 activity contributes to epithelial barrier dysfunction through modulation of Smad7 acetylation, though the precise molecular mechanisms warrant further investigation.

### Limitations of the study

Several limitations of this study should be acknowledged. First, while our Co-IP data demonstrate a physical association between HDAC8 and Smad7, we cannot definitively conclude that HDAC8 directly deacetylates Smad7. Second, the human PNECs used in this study were derived from a limited number of donors, and validation in a larger, independent cohort would strengthen the generalizability of our findings. Third, while the acetylation-mimetic Smad7 mutant (K64/70Q) attenuated HDAC8-induced effects, this approach mimics constitutive acetylation rather than directly demonstrating dynamic regulation. Future studies employing direct deacetylase assays, larger patient cohorts, and T-cell subset analyses will be necessary to address these limitations and further validate the proposed mechanism.

## Conclusion

In summary, this study provides evidence that HDAC8-mediated reduction of Smad7 acetylation may contribute to epithelial barrier dysfunction in AR. Our findings support a model in which HDAC8 interacts with Smad7 and is associated with decreased Smad7 acetylation and reduced TJ protein expression, effects that can be attenuated by HDAC8 inhibition. The observation that an acetylation-mimetic Smad7 mutant rescues HDAC8-induced TJ changes suggests that Smad7 acetylation status plays a role in this pathway. These findings identify HDAC8 as a potential therapeutic target for restoring mucosal homeostasis in AR, though further studies are needed to establish clinical applicability.

## Data Availability

The original contributions presented in the study are included in the article/**Supplementary Material**. Further inquiries can be directed to the corresponding authors.
