# Supplementary material for: HDAC8 impairs tight junctions in allergic rhinitis through Smad7 deacetylation
Source: Front Immunol. 2026 May 1;17:1793810. doi: 10.3389/fimmu.2026.1793810 (PMC13176201; doi:10.3389/fimmu.2026.1793810)
Supplement: Supplementary file 1 [file Table1.docx]

| Reagent/Antibody/Construct | Source | Catalog No. | Dilution/Concentration |
| --- | --- | --- | --- |
| Reagents | | | |
| PCI-34051 | Sigma-Aldrich | SML0449 | 50 μM (in vitro);  0.5 mg/kg (in vivo) |
| HDM extract | Greer Laboratories | XPB70D3A2.5 | 100 μg/mL (in vitro);  1 μg (sensitization);  10 μg/d (challenge); |
| Antibodies |  |  |  |
| Anti-HDAC8 | Cell Signaling Technology | 66042 | 1:1000 (WB); 2 μg (Co-IP) |
| Anti-Smad7 | Proteintech | 25840-1-AP | 1:1500 (WB); 2 μg (Co-IP) |
| Anti-ZO-1 | Cell Signaling Technology | 13663 | 1:1000 (WB); |
| Anti-Occludin | Cell Signaling Technology | 91131 | 1:1000 (WB) |
| Anti-Claudin-1 | Cell Signaling Technology | 13255 | 1:1000 (WB) |
| Anti-acetylated-lysine | Cell Signaling Technology | 9441 | 1:1000 (WB) |
| Anti-GAPDH | R&D Systems | MAB5718 | 1:1000 (WB) |
| **Plasmid Constructs** |  |  |  |
| pcDNA3.1-HDAC8 | This study | N/A | 2.5 μg/well |
| pcDNA3.1-Smad7-WT | This study | N/A | 2.5 μg/well |
| pcDNA3.1-Smad7-KQ | This study | N/A | 2.5 μg/well |

**Table S1. Key Reagents, Antibodies, and Constructs**

Note: All antibodies were used for Western blot (WB) unless otherwise specified. Co-IP, co-immunoprecipitation; HDM, house dust mite; KQ, acetylation-mimetic Smad7 mutant (K64/70Q); N/A, not applicable; WT, wild-type.
